# Sensitive Profiling of Human Milk Oligosaccharides in Human Colostrum and Breast Milk by Capillary Electrophoresis‐Mass Spectrometry

**DOI:** 10.1002/jssc.70309

**Published:** 2025-10-31

**Authors:** Denisa Smolkova, Marcelina Rusin, Justyna Dobrowolska‐Iwanek, Michał Woźniakiewicz, Jana Lavicka

**Affiliations:** ^1^ Institute of Analytical Chemistry of the Czech Academy of Sciences Brno Czech Republic; ^2^ Doctoral School of Exact and Natural Sciences Jagiellonian University Kraków Poland; ^3^ Department of Analytical Chemistry Faculty of Chemistry Jagiellonian University Kraków Poland; ^4^ Department of Food Chemistry and Nutrition Faculty of Pharmacy Jagiellonian University Medical College Kraków Poland

**Keywords:** breast milk, capillary electrophoresis, colostrum, human milk oligosaccharides, mass spectrometry

## Abstract

Human milk oligosaccharides are pivotal for shaping the infant gut microbiome and immune development, yet their structural diversity hampers routine identification and quantification. We report an optimized capillary electrophoresis–mass spectrometry workflow that enables sensitive, isomer‑selective profiling of 10 biologically relevant human milk oligosaccharides in colostrum and early‑lactation breast milk. Human milk oligosaccharides were first neutralized to stabilize sialic acids and derivatized with Girard's reagent P, introducing a permanent positive charge to enhance electrophoretic resolution and electrospray ionization efficiency. Separation in a linear‑polyacrylamide‑coated capillary (0.25 M formic acid, 30 kV) and mass spectrometry detection with a nanoCEasy interface achieved baseline resolution of all targets except positional isomers lacto‐*N*‐difucohexaose I/II. Incorporation of Girard's reagent P‑labeled maltoheptaose as an internal standard improved migration time precision to < 0.5% RSD and reduced peak‑area repeatability to 9%–25% RSD. Limits of detection were 0.8–290 ng/mL, corresponding to fg–pg on‑column amounts and outperforming precedent APTS‐based CE/LIF methodologies. Application to colostrum and milk samples from a single donor (1–3 months postpartum) revealed pronounced variation. Colostrum was dominated by 2′‑fucosyllactose and fucosylated lacto‐*N*‐fucopentaose isomers, whereas sialylated human milk oligosaccharides were present in smaller amounts. Longitudinally, 2′‑fucosyllactose remained the most abundant species, while lacto‐*N*‐fucopentaose and lacto‐*N*‐neotetraose/lacto‐*N*‐tetraose diminished markedly by Month 3. The presented capillary electrophoresis–mass spectrometry platform delivers reasonably fast (< 70 min), high‑sensitivity human milk oligosaccharide fingerprinting from minimal sample volumes and is readily adaptable to large‑cohort studies, offering new opportunities to elucidate the nutritional dynamics of the maternal milk glycome during lactation.

**Abbreviations**
2′FL: 2′‐fucosyllactose
3FL: 3‐fucosyllactose
3′SL: 3′sialyllactose
6′SL: 6′sialyllactose
APTS: 8‐aminopyrene‐1,3,6‐trisulfonic acid
BGE: background electrolyte
CE: capillary electrophoresis
DP7: maltoheptaose
DSLNT: disialyllacto‐*N*‐tetraose
EDC: *N*‐(3‐dimethylaminopropyl)‐*N*′‐ethylcarbodiimide
GP: Girard's reagent P
HBM: human breast milk
HMOs: human milk oligosaccharides
HOBt hydrate: 1‐hydroxybenzotriazole hydrate
ID: inner diameter
IS: internal standard
LNDFH I: lacto‐*N*‐difucohexaose I
LNDFH II: lacto‐*N*‐difucohexaose II
LNFP I: lacto‐*N*‐fucopentaose I
LNnT: lacto‐*N*‐neotetraose
LNT: lacto‐*N*‐tetraose
LPA: linear polyacrylamide
RSD: relative standard deviation

## 1 Introduction

Human breast milk (HBM) is a complex biological fluid that is the optimal source of nutrition for infants. HBM, being unique in its composition and nutritional value, provides essential ingredients for early growth and development, such as carbohydrates, proteins, fat, minerals, vitamins, as well as hormones, growth factors, and microbial communities. Among its diverse components, human milk oligosaccharides (HMOs) are the third largest component after lactose and fats in human milk [1, 2]. HMOs are complex glycans of five building units: glucose, galactose, *N*‐acetylglucosamine, fucose, and sialic acid. Among these units, glucose and galactose form the lactose core, constituting the reducing end, which additional building units can further elongate through glycosidic linkages. Currently, more than 200 different oligosaccharides have been identified in breast milk. With this, three main structural groups of HMOs have been distinguished: (i) neutral fucosylated (e.g., 2′‐fucosyllactose, [2′FL]), (ii) neutral‐core (e.g., lacto‐*N*‐tetraose, [LNT]), and (iii) acidic (e.g., 3′‐sialyllactose, [3′SL]) [3, 4, 5].

HMOs are compounds that resist low gastric pH and pancreatic digestive enzymes; therefore, after ingestion, they reach the distal areas of the digestive tract nearly unchanged. In the gut, they primarily provide nutrients that stimulate the growth of bifidogenic bacteria residing in the intestines [6]. Additionally, HMOs contribute to protecting the organism against bacterial, viral, and fungal infections through their anti‐adhesive and antimicrobial properties. HMOs also modulate epithelial cell responses by promoting protein expression and enhancing intestinal barrier function. However, it should be emphasized that 1% of oligosaccharides are absorbed and enter the systemic circulation, indicating their role in the functioning of other organs. The importance of HMOs and their metabolites, such as sialic acid, in brain development can be mentioned as an example. This widespread distribution implies that HMOs play a broader physiological role, impacting various aspects of neonatal health beyond the intestinal environment. Given the role that oligosaccharides play in the human body, they are undoubtedly one of the ingredients contributing to the proper development and health of newborns and infants. Deficiencies related to the lack of HMOs in their diet may have significant health consequences, such as an imbalanced gut microbiome or increased risk of infections, allergies, and inflammation [7, 8, 9].

It should be highlighted that analysis of HMOs is highly challenging because of their heterogeneity and diverse isomeric/anomeric structures. While liquid chromatography (LC) remains a more widely used technique for HMO analysis, capillary electrophoresis (CE) emerges as a valuable complementary approach, offering advantages such as high separation efficiency and low sample consumption [10]. A limitation of both CE and LC methods, when used without mass spectrometric (MS) detection, is the reliance on time‐based reference standards for compound identification. However, CE coupled to mass spectrometry (CE‐MS) has only rarely been applied to the analysis of HMOs. In the work of Zhong et al. [11], the CE‐MS was applied to examine the separation efficiency of aminooxy tandem mass tag‐labeled acidic HMO isomers, without direct analysis of milk samples. Only a few studies of Albrecht et al. [12, 13, 14] reported the analysis of 8‐aminopyrene‐1,3,6‐trisulfonic acid (APTS) labeled HMOs from breast milk and feces of breast‐fed babies using CE with laser‐induced fluorescence detection coupled to MS (CE/LIF‐MS). While APTS is commonly used for fluorescent labeling in CE due to its strong fluorescence and charge, it is not optimal for MS, as its ionization efficiency is limited. In contrast, Girard's reagent P (GP) introduces a permanent positive charge via a quaternary ammonium group, significantly enhancing ionization efficiency in a positive ion mode. To the best of our knowledge, no studies have applied GP labeling for CE‐MS analysis of HMOs from human milk, although this strategy has proven high sensitivity in the analysis of *N*‐linked glycans [15].

This paper focuses on analysis of 10 selected oligosaccharides from each of the main groups mentioned, which perform the most important functions in the infant's body and were indicated as particularly relevant in previous studies [4, 16, 17, 18]: 2′FL, 3‐fucosyllactose (3FL), 3′sialyllactose (3′SL), 6′sialyllactose (6′SL), lacto‐*N*‐difucohexaose I (LNDFH I), lacto‐*N*‐difucohexaose II (LNDFH II), lacto‐*N*‐fucopentaose I (LNFP I), LNT, lacto‐*N*‐neotetraose (LNnT) and disialyllacto‐*N*‐tetraose (DSLNT). Our approach utilizes derivatization by GP to enhance both the labeling efficiency and ionization of HMOs in CE‐MS analysis.

## 2 Materials and Methods

### 2.1 Chemicals and Materials

All chemicals for the derivatization and analysis were purchased from commercial vendors and used without further purification. Methanol, ethanol, acetonitrile, propan‐2‐ol, formic acid, acetic acid, sodium hydroxide, ammonium hydroxide, *N*‐(3‐dimethylaminopropyl)‐*N*′‐ethylcarbodiimide (EDC), 1‐hydroxybenzotriazole hydrate (HOBt hydrate), maltoheptaose (DP7), and GP were purchased from Merck (Prague, Czech Republic). HMO standards were purchased from DextraUK and Biosynth Carbosynth (90%–99%). Fused silica capillaries (50 µm ID, 365 µm OD) were purchased from Molex (Lisle, IL, USA). Amicon Ultra centrifugal filters (3 kDa MWCO) were also purchased from Merck.

In this study, colostrum and breast milk after 1, 2, and 3 months postpartum from one donor were collected at the University Hospital in Krakow, and after collection, the biological material was immediately frozen at −21°C. The study design was approved by the Bioethics Committee of the Jagiellonian University Medical College (No: 1072.6120.133.2018).

### 2.2 Breast Milk and Colostrum Sample Preparation

Breast milk and colostrum samples were prepared based on a previously reported method with slight modifications [19], which has been found to provide high recovery ranging from 89.3% to 110.29%. A volume of 100 µL of breast milk or colostrum was combined with an equal volume of ultrapure water. After thorough mixing, the mixture was centrifuged at 6000 rpm for 10 min at 4°C. Carefully avoiding the fatty upper layer, 100 µL of the aqueous phase was transferred into a clean test tube. Afterward, 200 µL of acetonitrile was added, followed by mixing and ultrasonic treatment for 5 min. The sample was then centrifuged at 10,000 rpm for 10 min at 4°C. From the resulting supernatant, 250 µL was collected, evaporated, and reconstituted in 450 µL of ultrapure water. Finally, the solution containing HMOs was filtered using Amicon filters (3 kDa MWCO), and the filtrate was dried before HMO labeling.

### 2.3 Sialic Acids Neutralization

The samples were processed following a previously described method with slight modifications [15]. Briefly, dried, processed samples of HMOs isolated from breast milk and colostrum or acidic HMOs standards were mixed with 20 µL of an ethyl esterification solution consisting of 0.25 M EDC and 0.25 M HOBt in ethanol. This mixture was incubated at 37°C for 1 h. Then, 4 µL of 28%–30% ammonium hydroxide was added to the reaction, followed by 2 h incubation at 37°C. The neutralized samples were dried and stored in the freezer before the further derivatization step.

### 2.4 Oligosaccharide Labeling by GP

HMOs (with/without a previous neutralization step used) were labeled by GP using a previously reported method [15], with some adjustments. HMO standard solutions (1–2 nanomoles each) were dried in a vacuum concentrator. Afterward, a labeling solution (50 µL) containing 0.01 M GP in methanol with 10% (v/v) acetic acid was added. The mixtures were incubated at 50°C for 18 h. After incubation, the samples were again dried using the vacuum concentrator and stored at −20°C until CE‐MS analysis.

This labeling approach was also applied to the labeling of human milk and colostrum samples, which had been prepared as described in Sections 2.2 and 2.3. However, the labeling proceeded in a solution (50 µL) containing 0.2 M GP in methanol with 10% (v/v) acetic acid.

### 2.5 CE‐MS Analysis

All CE‐MS analyses were performed using an Agilent 7100 CE system (Waldbronn, Germany) coupled with a maXis Impact ESI‐TOF mass spectrometer (Bruker Daltonics, Bremen, Germany) via the nanoCEasy interface [20]. The capillary for CE separations with a 50 µm inner diameter (ID) and an effective length of 80 cm or 100 cm was coated with neutral linear polyacrylamide (LPA). The LPA coating procedure was adapted from the published protocol [21]. The LPA‐coated capillary was preconditioned by rinsing with water for 15 min, followed by a 10‐min rinse with the background electrolyte (BGE) at a pressure of 1 bar. The separations were performed in a BGE consisting of 0.25 M formic acid or 1 M acetic acid. Samples were dissolved in ultrapure water with GP‐labeled DP7 as an internal standard (IS). The samples were injected at a pressure of 50 mbar for 10 or 15 s, followed by the injection of a BGE plug at 50 mbar for 5 s. All separations were performed at laboratory temperature with an applied voltage of +30 kV. Between analyses, the capillary was flushed with the separation buffer at 1 bar for 10 min to prevent cross‐contamination.

For ESI, a sheath liquid consisting of 1% (v/v) formic acid and 50% (v/v) propan‐2‐ol was supplied at a flow rate of 6.5 µL/min using a syringe pump, although only around 100 nL/min reached the emitter tip [20]. The nanoCEasy system setup and the emitter tip alignment, approximately 5 mm from the MS inlet, were monitored using a digital microscope. Electrospray ionization was performed in a positive mode using an ESI voltage of +2.0 kV. Data acquisition occurred at a scan rate of 1 Hz across a mass range of 400–2000 *m*/*z*. Identification of the labeled compounds was based on the accurate mass of singly charged ions, with a mass tolerance of ±0.05 Da. Results are presented as extracted ion electropherograms (EIEs) of individual labeled HMOs.

## 3 Results and Discussion

Despite the majority of HPLC‐based methods, CE and mainly CE‐MS can represent a valuable complementary approach for the analysis of HMOs. To enhance the electrophoretic mobilities of HMOs, efficient labeling using GP was employed prior to CE‐MS analysis [15]. This derivatization strategy introduced a permanent positive charge to the oligosaccharide molecules, thereby allowing electrophoretic separation as well as enhancing ionization efficiency and MS detection. GP labeling is based on hydrazone formation chemistry, providing a high reaction yield under mild reaction conditions [22, 23]. It has been shown that the labeling via hydrazone formation chemistry provides a reaction yield of ∼90%, which is almost one order of magnitude higher compared to reductive amination [23]. In this work, we have focused on the analysis of 10 HMOs present in HBM and colostrum, namely 2′FL, 3FL, 3′SL, 6′SL, LNDFH I, LNDFH II, LNFP I, LNT, LNnT, and DSLNT. The structures of individual HMOs are gathered in Table 1.

**TABLE 1:** Analyzed HMOs and corresponding m/z values of GP‐labeled HMOs.

HMOs			
Name	Abbreviation	Structure	*m*/*z*
3‐fucosyllactose	3FL	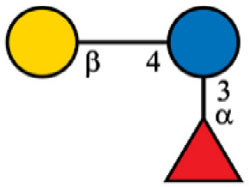	622.34
2′‐fucosyllactose	2′FL	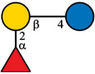	622.34
6′‐sialyllactose	6′SL	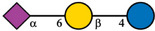	767.38, 795.41 (neutralized sialic acid)
3′‐sialyllactose	3′SL	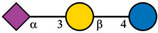	767.38, 766.39 (neutralized sialic acid)
Lacto‐*N*‐tetraose	LNT	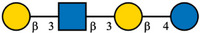	841.41
Lacto‐*N*‐neotetraose	LNnT	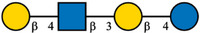	841.41
Lacto‐*N*‐fucopentaose I	LNFP I	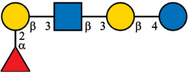	987.47
Lacto‐*N*‐difucohexaose I	LNDFH I	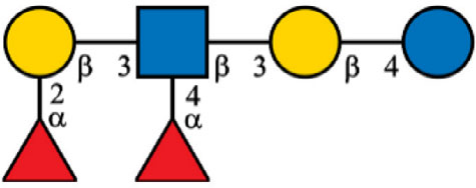	1133.53
Lacto‐*N*‐difucohexaose II	LNDFH II	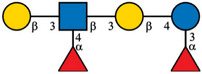	1133.53
Disialyllacto‐*N*‐tetraose	DSLNT	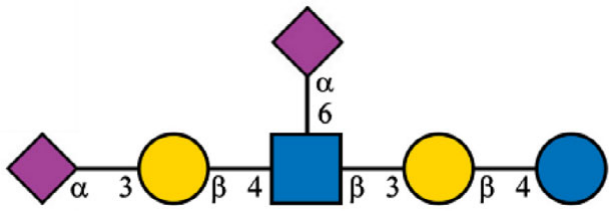	1423.61, 1450.65 (neutralized sialic acids)

*Note*: 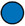 glucose, 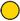 galactose, 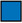 *N*‐acetylglucosamine, 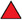 fucose, 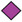 *N*‐acetylneuraminic acid.

### 3.1 Optimization of CE‐MS Analysis of GP‐Labeled HMOs

**FIGURE 1:**
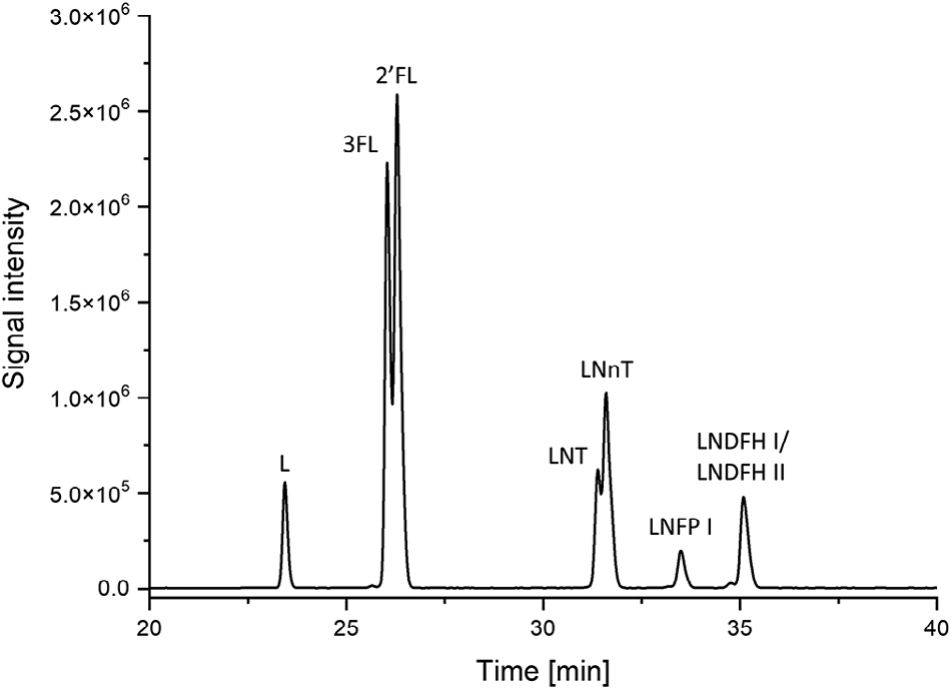
CE‐MS electropherogram (EIE) of the GP‐labeled HMO standard mixture. Experimental conditions: LPA‐coated capillary (50 µm ID, 80 cm), BGE: 1 M acetic acid, sheath liquid: 1% (v/v) formic acid and 50% (v/v) propan‐2‐ol, sample concentration: 20 µg/mL each oligosaccharide, sample injection: 50 mbar, 15 s, separation voltage: 30 kV, ESI voltage: 2.0 kV.

The analytical procedure was optimized through the use of a mixture of 10 GP‐labeled HMO standards. To prevent acidic HMOs degradation and the loss of sialic acid during the derivatization at elevated temperature, the labeling process was conducted only at 50°C for a longer reaction time (18 h), employing an excess of the labeling reagent to ensure complete derivatization. Subsequently, the GP‐labeled HMOs were analyzed using CE‐MS with the nanoCEasy interface. The separation was performed in the 50 µm ID LPA‐coated capillary (80 cm long) and 1 M acetic acid as BGE. A solution consisting of 1% (v/v) formic acid and 50% (v/v) propan‐2‐ol was used as a sheath liquid in CE‐MS coupling. As shown in Figure 1, these separation conditions provided an efficient separation of neutral/fucosylated HMOs. Despite the partial separation of fucosylated lactose isomers (2′FL and 3FL) as well as LNT and LNnT, no separation of LNDFH I and LNDFH II isomers was observed. Due to the permanent charge associated with the GP tag, the labeled HMOs were primarily detected and identified as singly charged species. The *m*/*z* values of the GP‐labeled HMOs are summarized in Table 1. However, no acidic oligosaccharides were identified within the 60‐min separation time. Because the label only confers a single positive charge, the migration times for 3′SL and 6′SL extended up to 100 min, and DSLNT, which contains two sialic acid molecules, was not detected at all under these separation conditions. Consequently, a derivatization step was incorporated into the sample preparation procedure to neutralize the sialic acids by amidation. This complementary derivatization also improved the stability of acidic HMO during the MS analysis.

**FIGURE 2:**
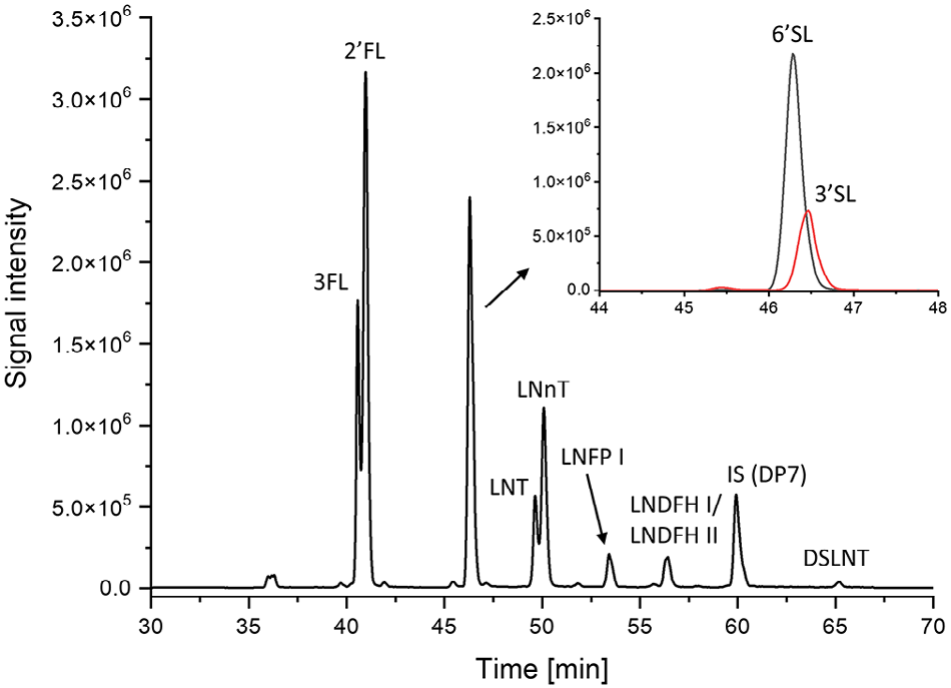
CE‐MS electropherogram (EIE) of the GP‐labeled HMO standard mixture. Experimental conditions: LPA‐coated capillary (50 µm ID, 100 cm), BGE: 0.25 M formic acid, sheath liquid: 1% (v/v) formic acid and 50% (v/v) propan‐2‐ol, sample concentration: 20 µg/mL each oligosaccharide, sample injection: 50 mbar, 10 s, separation voltage: 30 kV, ESI voltage: 2.0 kV.

Additionally, a BGE composition was optimized in order to improve separation efficiency. Due to the limitations for MS compatibility, only different concentrations of acetic and formic acids were evaluated to optimize CE‐MS conditions. Although the BGE composition primarily affects separation efficiency, it also influences electrospray stability. The best results were obtained with 0.25 M formic acid as BGE, which also provided a more stable electrospray and consistent MS signals across multiple runs. It should also be noted that the longer (100 cm long) separation capillary was used in the following CE‐MS analyses. EIEs of GP‐labeled HMOs with neutralized sialic acids are shown in Figure 2. These derivatization and separation conditions enabled near baseline separation of all HMO isomers relevant to infant health, with the exception of LNDFH I and LNDFH II. Even after thorough optimization of the separation conditions, these two isomers could not be resolved and remained indistinguishable under the current method. Nevertheless, their limited biological significance compared to other HMOs reduces the overall negative impact of this limitation on the analytical outcome. However, the neutralization step allowed for the detection and identification of all three acidic HMOs investigated. Although 3′SL and 6′SL were detected at almost the same time (46 min), the implemented treatment enabled the differentiation of these two positional isomers, based on distinct mass shifts observed in their neutralized forms [24]. The α2,6‐linked sialic acid in 6′SL forms a stable ethyl ester when treated with EDC/HOBt in ethanol, resulting in a mass increase of 28.031 Da. On the other hand, the α2,3‐linked sialic acid in 3′SL undergoes lactonization followed by ring opening and amidation in the presence of ammonium hydroxide. This reaction leads to a mass decrease of 0.984 Da. The m/z values of neutralized and GP‐labeled acidic HMOs are listed in Table 1. The corresponding mass shifts were also observed in the case of the DSLNT oligosaccharide (peak in 65 min), containing both α2,3‐ and α2,6‐linked sialic acids in the structure.

### 3.2 Evaluation of CE‐MS Analysis of GP‐Labeled HMOs

The presented CE‐MS method were characterized in terms of repeatability and detection sensitivity. The repeatability of migration times and peak areas were expressed as relative standard deviation (RSD), calculated from five repeated runs of GP‐labeled HMO standard CE‐MS analyses. Although the repeatability of CE‐MS migration times was found to be below 1% RSD, the repeatability of peak areas did not meet the required criteria, with the values exceeding 30% RSD for most HMOs analyzed. This can be attributed mainly to the open setup of the CE‐MS interface. The sheath liquid dilutes the separated zone of the analytes entering the emitter tip, and this dilution is very sensitive to the experimental conditions, such as the flow rate of the sheath liquid and ESI voltage [25]. Therefore, the CE‐MS analyses were performed with IS added to the labeled samples. DP7, also labeled by GP, was selected as IS due to its commercial availability and a migration time occurring shortly before the slowest HMO standard (DSLNT), making it well‐suited for accurate recalculation of peak areas. The results shown in Table 2 confirmed the importance of using IS, particularly for the improvement of the peak area repeatability. RSDs of migration times ranged from 0.2% to 0.5% with IS and 0.5% to 0.7% without IS. More importantly, RSDs of peak areas showed a significant improvement in repeatability by decreasing RSD values from 28% to 45% without IS to 9% to 25% with IS. The limits of detection (LODs) of individual HMOs were determined to be 0.8–290 ng/mL (S/N = 3, *n* = 5) as shown in Table 2. The values reflect the fg‐pg amounts of injected HMOs. The low signal observed for DSLNT, along with the corresponding higher LOD, may be attributed to incomplete neutralization of the sialic acids, resulting only in a partial detection of this HMO despite a well‐established derivatization protocol [15]. Furthermore, the long migration time of GP‐labeled DSNLT, leading to peak broadening, could also be eliminated by a proper optimization of BGE, for example, reversing the migration order and decreasing the overall time of analysis [26, 27]. Nonetheless, the overall high sensitivity of the CE‐MS analysis can be ascribed to the derivatization of HMOs by positively charged GP through hydrazone formation chemistry, as well as the execution of CE‐MS analysis in the positive ion mode using the highly sensitive nanoCEasy interface. In comparison, the CE/LIF analysis of HMOs provided the LOD of 0.025 mg/mL (S/N = 3.71) as determined for APTS‐labeled para‐lacto‐*N*‐neohexaose [28] or 1–4 ng/mL (S/N = 3) for 2‐AMAC‐labeled HMOs [29].

**TABLE 2:** Evaluation of the CE‐MS analysis of GP‐labeled HMOs.

HMO	Repeatability of migration time (RSD, %), without IS	Repeatability of migration time (RSD, %), with IS	Repeatability of peak area (RSD, %), without IS	Repeatability of peak area (RSD, %), with IS	LODs (ng/mL)
3′FL	0.6	0.5	36.7	25.8	1.9
2′FL	0.6	0.5	28.3	21.9	0.8
6SL	0.5	0.4	32.5	17.9	1.3
3SL	0.6	0.4	36.9	14.1	4.2
LNT	0.6	0.3	34.2	12.0	8.7
LNnT	0.6	0.3	36.2	13.7	3.9
LNFP I	0.6	0.2	45.2	21.5	26.4
LNDFH I/II	0.6	0.2	30.1	10.6	26.5
DSLNT	0.7	0.2	29.8	9.2	290.0

### 3.3 CE‐MS Analysis of GP‐Labeled HMOs in Colostrum and Mature Breast Milk

The neutralization step and GP labeling, followed by the developed CE‐MS method, were also applied to the analysis of HMOs in colostrum and breast milk samples. In addition to the colostrum sample collected at the University Hospital in Krakow, every month during early lactation (initial 180 days), samples of mature breast milk were obtained from the same donor to monitor changes in selected HMOs during lactation.

Breast milk contains significant amounts of lipids and proteins, in addition to HMOs and lactose. Thus, it was essential to remove these compounds from the samples prior to further analysis. The lipid fraction was separated through centrifugation, followed by the precipitation of proteins using acetonitrile. This was followed by centrifugation and subsequent filtration to eliminate the proteins. Similar to the analysis of HMO standards, the acidic HMOs found in colostrum and breast milk were neutralized before proceeding with GP labeling of all HMOs. Prior to sample injection, IS (GP‐labeled DP7) was added to the GP‐labeled samples. Given the substantial presence of lactose in both colostrum and mature breast milk, it was crucial to minimize its interference during MS detection. This challenge was effectively addressed using the nanoCEasy interface, which allows for the manual repositioning of the separation capillary in the glass emitter during lactose migration, moving it far from the emitter tip. As a result, excess lactose was directed to waste, thereby preventing its entry into the MS.

**FIGURE 3:**
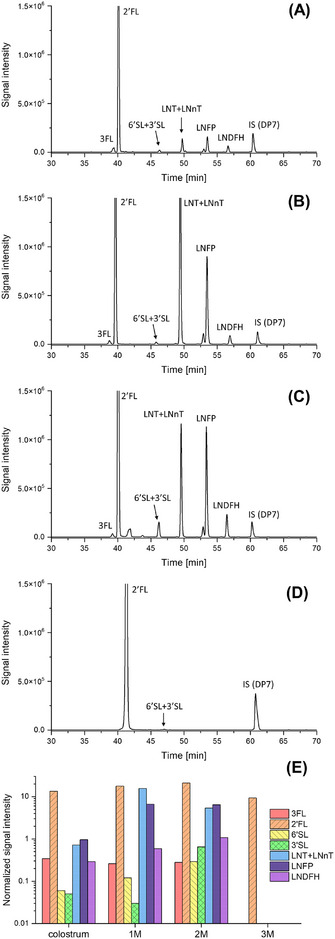
CE‐MS electropherograms (EIEs) of the GP‐labeled HMO in (A) colostrum and breast milk (B—1‐month lactation, C—2‐month lactation, D—3‐month lactation) samples from donor D3 and (E) corresponding quantitative changes in individual HMOs (normalized to the signal of an internal standard). Experimental conditions: LPA‐coated capillary (50 µm ID, 100 cm), BGE: 0.25 M formic acid, sheath liquid: 1% (v/v) formic acid and 50% (v/v) propan‐2‐ol, sample dilution: 67×, sample injection: 50 mbar, 10 s, separation voltage: 30 kV, ESI voltage: 2.0 kV.

Figure 3 shows EIEs of GP‐labeled HMOs signals collected during the analysis of colostrum and milk samples collected from a single volunteer (D3) during the distinct phases of early lactation, employing a sample dilution ratio of 67×: colostrum (A), and milk from the first (B), second (C), and third (D) month postpartum. The identification of the GP‐labeled HMOs was accomplished through the analysis of *m*/*z* values and migration times. The developed method enabled effective detection of HMOs in real, non‐standardized milk samples, demonstrating its practical applicability. The presence of multiple oligosaccharides was confirmed across all analyzed timepoints, with 2′FL being consistently observed in each sample with the highest signal intensity. Differences in the HMOs’ signal intensities were observed between the samples collected at various lactation stages, including a reduced number of detected compounds in the third month postpartum. The quantitative changes in individual HMOs during the 3‐month lactation period are summarized in Figure 3E. These preliminary findings from our study using the CE‐MS approach are in good agreement with previously published papers [30, 31, 32, 33], demonstrating that HMO profiles undergo changes throughout different stages of lactation. These changes include variations in total HMO concentration, the composition of specific HMO groups, and overall diversity. For example, it has been shown that 2′FL can constitute up to 30% of the total HMO pool during early lactation, particularly in secretor‐positive mothers. In addition to 2′FL, other major HMOs detected in colostrum and milk samples included fucosylated isomers (like LNFP) and neutral‐core HMOs (LNT/LNnT). Different forms of LNFP show distinct trends depending on both the lactation stage and maternal secretor status. The levels of LNFP generally increase during lactation and later show a consistent decrease from colostrum to mature milk. Our findings are consistent with these general trends reported in the literature, where the oligosaccharide profile in colostrum is dominated by fucosylated structures, which often account for more than 50% of HMOs’ total content. On the other hand, the concentration of sialylated HMO, such as 3′SL and 6′SL, tends to increase during the early stages of lactation and then decrease in mature milk.

Therefore, HMO profiles are highly dynamic and vary considerably not only with lactation stage, but also other factors, including maternal secretor status. This highlights the complexity of HMO composition and underscores that our analytical method is well‐suited for the sensitive and detailed profiling of HMOs in complex milk matrices. Nevertheless, it should be emphasized that this study is limited in scope, focusing on a single donor. Therefore, while the observed trends are in good agreement with existing data, the results should not be interpreted as broadly representative. Further research involving an extensive cohort study is required to validate these preliminary observations and to better understand interindividual and temporal variability in HMO profiles. The successful application of the developed methodology to real milk samples highlights the reliability of the approach. Thus, while limited in scope, the present study provides a valuable proof‐of‐concept for sensitive and detailed HMO profiling in complex biological matrices.

## 4 Concluding Remarks

We successfully optimized a CE‐MS analytical approach for the analysis of the most biologically relevant HMOs in colostrum and mature breast milk samples. Samples were derivatized using sialic acid neutralization followed by GP labeling. The incorporated neutralization step allowed distinguishing acidic HMOs, 3′SL and 6′SL, and the detection of DSLNT, bearing two sialic acids in the structure, in the positive MS mode. Despite the unsuccessful separation of LNDFH I and LNDFH II positional isomers, the labeling based on hydrazone formation chemistry, characterized by a high reaction yield, provided high sensitivity of CE‐MS analysis of labeled HMOs with LODs nearly comparable or even better than that obtained by the CE/LIF approach.

Only a few studies have applied CE‐MS to the analysis of HMOs. A notable example is the work of Albrecht et al. [13], who combined CE/LIF and online coupling to MS*ⁿ*, enabling broad profiling and structural elucidation of HMOs in human milk and infant feces. This CE/LIF‐MS*ⁿ* approach provided high‐resolution separation and structural assignment of multiple isomers, but required fluorescence labeling with APTS and extensive MS*ⁿ* experiments. In contrast, our method was designed as a targeted CE‐MS workflow for the quantification of 10 selected HMOs. By employing GP derivatization and sialic acid neutralization, we achieved robust and sensitive detection directly in MS mode, without the need for additional LIF detection or multi‐stage fragmentation.

It should be noted that the concentration as well as the composition of HMOs vary from individual to individual and from lactation to lactation. In addition to genetic factors, health and other maternal factors, such as body weight, nutritional intake, and age, affect the composition of colostrum and breast milk, including HMOs to some extent [3, 4]. Therefore, to draw robust conclusions about changes in HMO profiles and to explore potential correlations with maternal conditions such as diabetes, a more extensive, statistically powered study including a larger number of samples will be necessary.

## Author Contributions

**Denisa Smolkova**: investigation; visualization; writing – original draft. **Marcelina Rusin**: investigation; formal analysis; writing – original draft. **Justyna Dobrowolska‐Iwanek**: writing – review and editing; resources. **Michał Woźniakiewicz**: writing – review and editing. **Jana Lavicka**: conceptualization; supervision; funding acquisition; project administration; writing – review and editing.

## Conflicts of Interest

The authors declare no conflicts of interest.

## Data Availability

The data that support the findings of this study are openly available in the ASEP Data Repository at https://doi.org/10.57680/asep.0637285, reference number 0637285.
